# Detection of Pathological Markers of Neurodegenerative Diseases following Microfluidic Direct Conversion of Patient Fibroblasts into Neurons

**DOI:** 10.3390/ijms23042147

**Published:** 2022-02-15

**Authors:** Cristiana Mollinari, Chiara De Dominicis, Leonardo Lupacchini, Luigi Sansone, Davide Caprini, Carlo Massimo Casciola, Ying Wang, Jian Zhao, Massimo Fini, Matteo Russo, Enrico Garaci, Daniela Merlo

**Affiliations:** 1 Department of Neuroscience, Istituto Superiore di Sanita’, 00161 Rome, Italy; cristiana.mollinari@ift.cnr.it (C.M.); chiaradedominicisjob@gmail.com (C.D.D.); 2 Institute of Translational Pharmacology, National Research Council, 00133 Rome, Italy; 3 Technoscience, 00166 Rome, Italy; 4 IRCCS San Raffaele Roma, 00163 Rome, Italy; leonardo.lupacchini@sanraffaele.it (L.L.); luigi.sansone@sanraffaele.it (L.S.); massimo.fini@sanraffaele.it (M.F.); matteo.russo@sanraffaele.it (M.R.); garenco@icloud.com (E.G.); 5 Center for Life Nano and Neuro Sciences (CLN2S@Sapienza), Istituto Italiano di Tecnologia, 00161 Rome, Italy; davide.caprini@iit.it; 6 Department of Mechanical and Aerospace Engineering, Sapienza University of Rome, 00184 Roma, Italy; carlomassimo.casciola@uniroma1.it; 7 Lab of Regenerative Medicine and Neural Repair, Shanghai Institute for Advanced Immunochemical Studies and School of Life Science and Technology, ShanghaiTech University, Shanghai 201210, China; wangying1@shanghaitech.edu.cn (Y.W.); zhaojian@shanghaitech.edu.cn (J.Z.); 8 MEBIC Consortium, San Raffaele University, 00166 Rome, Italy; 9 San Raffaele Roma Open University, 00166 Rome, Italy

**Keywords:** transdifferentiation, induced neurons, neurodegeneration, biomarkers, microfluidics

## Abstract

Neurodegenerative diseases such as Alzheimer’s disease and Parkinson’s disease are clinically diagnosed using neuropsychological and cognitive tests, expensive neuroimaging-based approaches (MRI and PET) and invasive and time-consuming lumbar puncture for cerebrospinal fluid (CSF) sample collection to detect biomarkers. Thus, a rapid, simple and cost-effective approach to more easily access fluids and tissues is in great need. Here, we exploit the chemical direct reprogramming of patient skin fibroblasts into neurons (chemically induced neurons, ciNs) as a novel strategy for the rapid detection of different pathological markers of neurodegenerative diseases. We found that FAD fibroblasts have a reduced efficiency of reprogramming, and converted ciNs show a less complex neuronal network. In addition, ciNs from patients show misfolded protein accumulation and mitochondria ultrastructural abnormalities, biomarkers commonly associated with neurodegeneration. Moreover, for the first time, we show that microfluidic technology, in combination with chemical reprogramming, enables on-chip examination of disease pathological processes and may have important applications in diagnosis. In conclusion, ciNs on microfluidic devices represent a small-scale, non-invasive and cost-effective high-throughput tool for protein misfolding disease diagnosis and may be useful for new biomarker discovery, disease mechanism studies and design of personalised therapies.

## 1. Introduction

Neurodegenerative diseases are increasing in prevalence and have a devastating impact on the affected individuals and their families. There are no effective treatments for the great majority of patients, and the primary disease causes are unknown, except in a small number of familial cases driven by genetic mutations [1,2].

Alzheimer’s disease (AD) and Parkinson’s disease (PD) are clinically diagnosed by physical and neurological examinations as well as standard neuropsychological and cognitive tests. Biomarkers like β-Amyloid, α-Synuclein and phospho-TAU (P-TAU) are crucial for diagnostic accuracy and are considered the most indicative of AD and PD pathology [3].

Cerebrospinal fluid (CSF) represents a logical source for developing viable biomarkers, given its direct interaction with the extracellular space in the brain, thus potentially reflecting the associated pathophysiological alterations. However, the lumbar puncture procedure for CSF sample collection is time consuming, invasive and not widely available. Among the molecular imaging biomarkers, PET detecting β-Amyloid and P-TAU has the significant advantage of low invasiveness, but the high cost represents its main limitation, and its generalised use is not yet feasible in all clinical settings [4,5].

Direct conversion of human skin fibroblasts into induced neurons represents an innovative strategy to overcome inaccessibility of the human brain to obtain human neurons for modelling neurodegenerative disorders, mechanistic studies and drug screening [6,7]. Direct conversion of fibroblasts into neuronal cells, a process called transdifferentiation, can be induced by lineage-specific transcription factors (induced neurons, iNs) or chemical cocktails (chemically induced neurons, ciNs). Interestingly, it has been shown that the induced neurons can maintain the features imparted by aging, and disease progression is not erased or altered [7,8].

Recent advances in cell biology, microfabrication and microfluidics have enabled the development of micro-engineered models offering the possibility of managing biological samples in tiny channels and chambers (chips) [9]. The microfluidic lab-on-chip platform, used for the validation of neuronal induction protocols, significantly reduces the costs of reagents, including cells, culture media and drugs, allowing high-throughput experiments that are not economically sustainable at a macroscopic level. Thus, the innovative approach based on the use of microfluidics combined with the transdifferentiation of patient-specific somatic cells into neurons is emerging as a powerful technology, aiding in the implementation of personalised medicine aimed at diagnosis and therapy [7,10].

The aim of the present study was to establish a new on-chip human cell–based assay for the diagnosis of neurodegenerative diseases through the detection of neuropathological markers. To this aim, altered neuronal morphology, β-Amyloid and α-Synuclein accumulation, TAU phosphorylation and mitochondrial abnormalities were investigated in ciNs derived from fibroblasts of healthy subjects, patients with familial AD (FAD/PSEN1) and patients with PD.

## 2. Results

### 2.1. Chemical Reprogramming of Human Dermal Fibroblasts into Neurons from Healthy Subjects and FAD Patients

**Figure 1:**
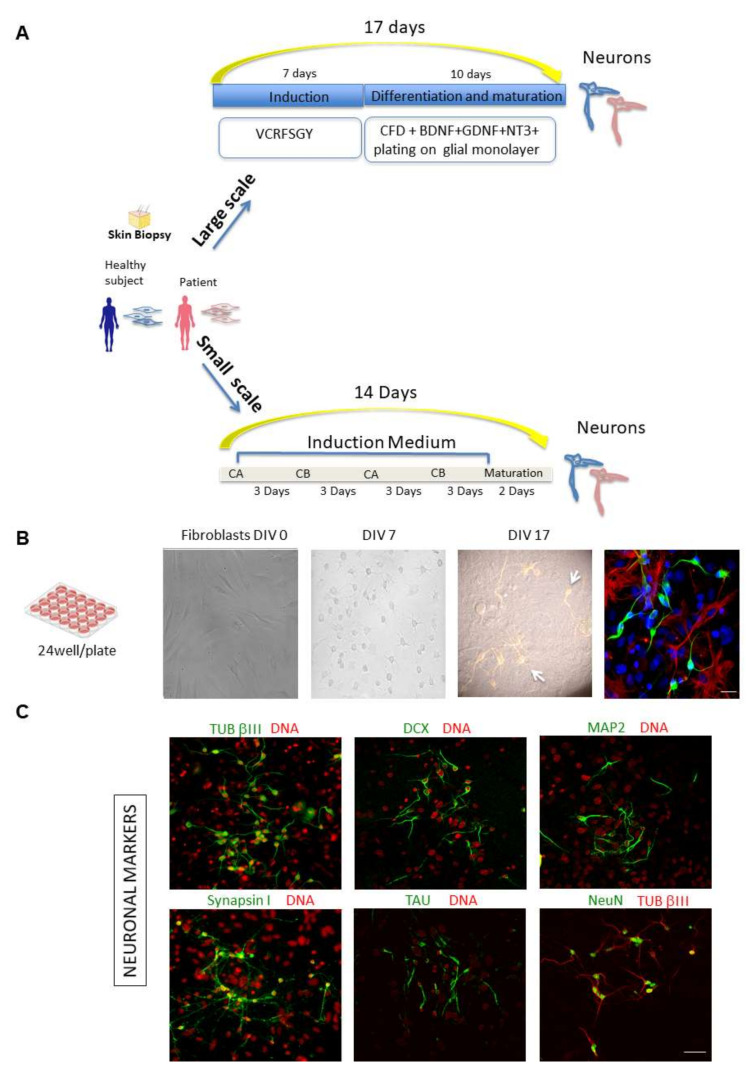
Chemical approaches to covert human fibroblasts into neurons: large-scale and small-scale experiments. (**A**) Schematic representation of the chemical approaches used to covert fibroblasts from healthy subjects and patients into neurons. Human fibroblasts isolated from skin biopsy (from Coriell BioBank, Camden, NJ, USA) were treated with specific chemical cocktails. Large scale: Fibroblasts were plated in DMEM in a 24-well plate (DIV 0). One day later, cells were transferred into induction medium with chemical compounds VCRFSGY (V, VPA; C, CHIR99021; R, Repsox; F, Forskolin; S, SP600625; G, GO6983; Y, Y-27632) and cultured for 7 days. Cells were then detached by enzymatic activity, further re-plated onto a layer of mouse glial cells and cultured in maturation medium with supplementary chemicals CFD (C, CHIR99021; R, Repsox; F, Forskolin; D, Dorsomorphin) and neurotrophic factors for 10 days. After fixation and permeabilization, cells were subjected to confocal microscopy analysis. Small scale: Schematic representation of the procedure for direct conversion of fibroblasts using chemical compounds on chip devises. A combination of medium CA, which mainly inhibits fibroblast signalling cascade and initiates neuronal fate transformation, in alternance with medium CB, which maintains cell survival and enhances reprogramming efficiency, is used to obtain the direct reprogramming towards a neuronal phenotype on chip. (CA: containing CHIR99021, LDN193189, RG108, dorsomorphin, P7C3-A20, A83-01, and ISX9); (CB: containing Forskolin, Y27632, DAPT, PD0325901, A83-01, purmorphamine, and P7C3-A20). (**B**) Representative phase-contrast images of a large-scale experiment to chemically convert human control fibroblasts showing a time course of cells at DIV 0, 7 and 17. At DIV 17, TUB βIII positive cells form a network on top of the glial monolayer. A representative composite confocal image of differentiating ciNs (DIV12) is shown by labelling with anti-TUB βIII antibody (green channel), laying on a layer of glial cells labelled with an anti-GFAP antibody (red channel). Nuclei are counterstained with Hoechst (blue channel). Scale Bar, 20 μm. (**C**) Representative double immunofluorescence images from a large-scale experiment show that ciNs express typical early and late neuronal markers, including TUB βIII, DCX, MAP2, Synapsin I, NeuN and TAU. Scale Bar, 50 μm.

We exploited direct conversion of human dermal fibroblasts using exclusively small molecules as a tool to obtain human neurons that, when plated on a glial monolayer, express typical neuronal markers and display electrophysiological properties (Figure 1A,B) [11,12]. Indeed, by confocal microscopy we detected both early and late markers of neuronal differentiation, including Tubulin βIII (TUB βIII), Doublecortin (DCX), Microtubule associated protein 2 (MAP2), Synapsin I, TAU and Neuronal Nuclear Protein (NeuN) (Figure 1C).

Fibroblasts from healthy subjects and from patients with known phenotypes such as FAD/PSEN1 and PD (Table 1) were obtained from the Coriell Institute Biobank (Camden, NJ, USA) and used to perform both large-scale (24-well plates) and small-scale (microfluidic chips) experiments (Figure 1A).

**Figure 2:**
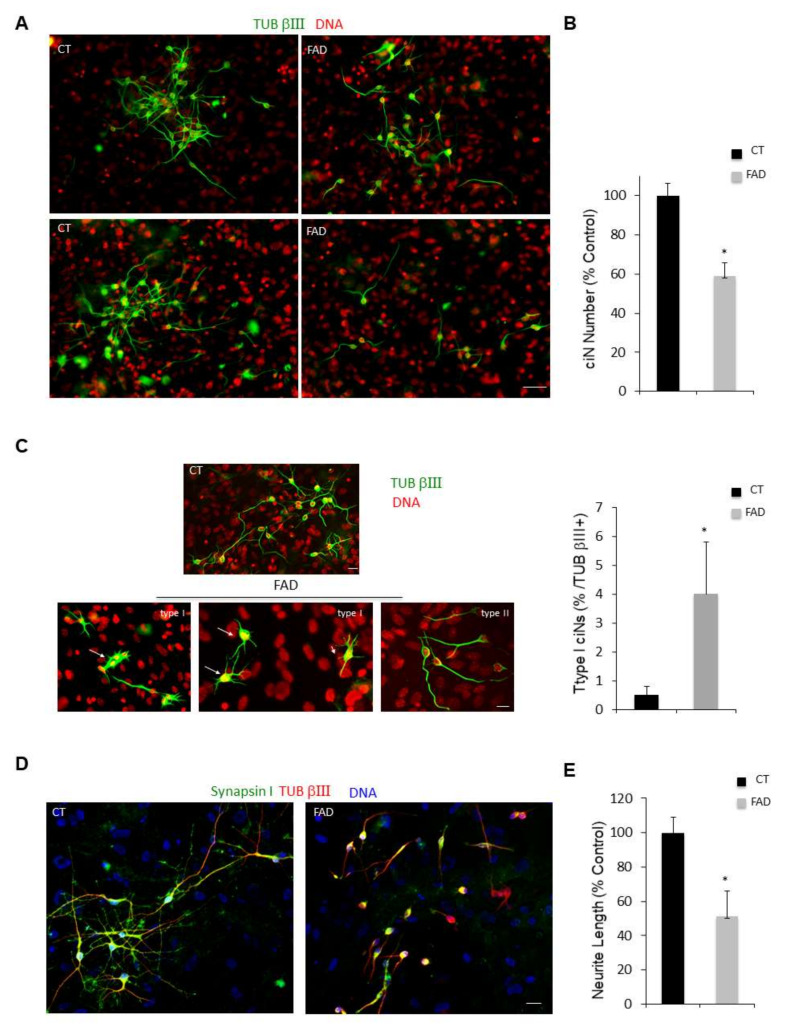
Patient fibroblasts have a reduced efficiency of reprogramming and patient ciNs present a less complex neuronal network. (**A**) Immunofluorescence images of ciNs obtained from direct conversions of healthy subject (CT) and Alzheimer’s Disease (FAD) patient fibroblasts, labelled with the neuronal marker TUB βIII (green channel) and DNA (red channel). Representative images show that FAD fibroblasts can be converted into neurons with a lower efficiency as compared with CT fibroblasts. Scale Bar, 50 μm. (**B**) Histogram data represent the percent change in the number of ciNs of FAD fibroblasts with respect to control fibroblasts. Bars in the plots represent means ± SD (n = 3, * *p* < 0.05 vs. control values). (**C**) Confocal fluorescence images show that following FAD fibroblast chemical conversion, two types of TUB βIII-positive ciNs are generated with different morphology. The so-called “type I” ciNs have flat body and a high number of short protrusions, whereas “type II” cells have a morphology similar to CT ciNs and present small cellular bodies with two or three neurites of variable length (TUB βIII, green channel; DNA red). Scale Bar, 10 μm. Histogram data show the percentage of Type I cells with respect to total TUB βIII + cells. (**D**) Analysis of neurite length of “type II” ciNs labelled with anti-Synapsin I antibody (a presynaptic marker, green channel), TUB βIII (red) and DNA (blue) show that FAD ciNs have a lower neurite complexity and a reduced neurite length as compared with CT cells. Scale Bar, 20 μm. (**E**) Histogram data represent the percent change in neurite length in FAD ciNs with respect to control neurons. Bars in the plots represent means ± SD (n = 3, * *p* < 0.05 vs. control values).

We first performed large-scale experiments by seeding fibroblasts on Matrigel-coated 24-well plates or glass coverslips to carry out a careful characterisation of ciNs [11] and to compare ciNs from healthy and FAD fibroblasts (Figure 2). We found that FAD fibroblasts show a reduced conversion efficiency as compared with healthy fibroblasts (41% reduction in FAD ciNs: FAD 59 ± 6.5% expressed as % of control) (Figure 2A,B). Moreover, immunostainings performed to characterise the morphological phenotype of ciNs from healthy and FAD fibroblasts revealed that FAD ciNs show an altered neuronal morphology (Figure 2A,C and Figure S1). In particular, as previously reported by us and others, as a result of oxidative damage [13,14], FAD ciNs generate two different types of TUB βIII-positive cells: the so-called “type I” cells, showing large, flat somata with multiple short processes (4 ± 1.8% with respect to the total FAD cells acquiring the neuronal phenotype), and “type II” cells, showing small, round somata with two to three long processes (Figure 2C). Furthermore, ciNs from FAD fibroblasts present a less complex neuronal network in type II cells and show a 49% reduction in neurite length (FAD 51 ± 15%, expressed as % of control) (Figure 2D,E), allowing a first gross distinction between healthy and patient cells.

### 2.2. Detection of Neuropathologicak Markers in ciNs Neurons from FAD and PD Patients

**Figure 3:**
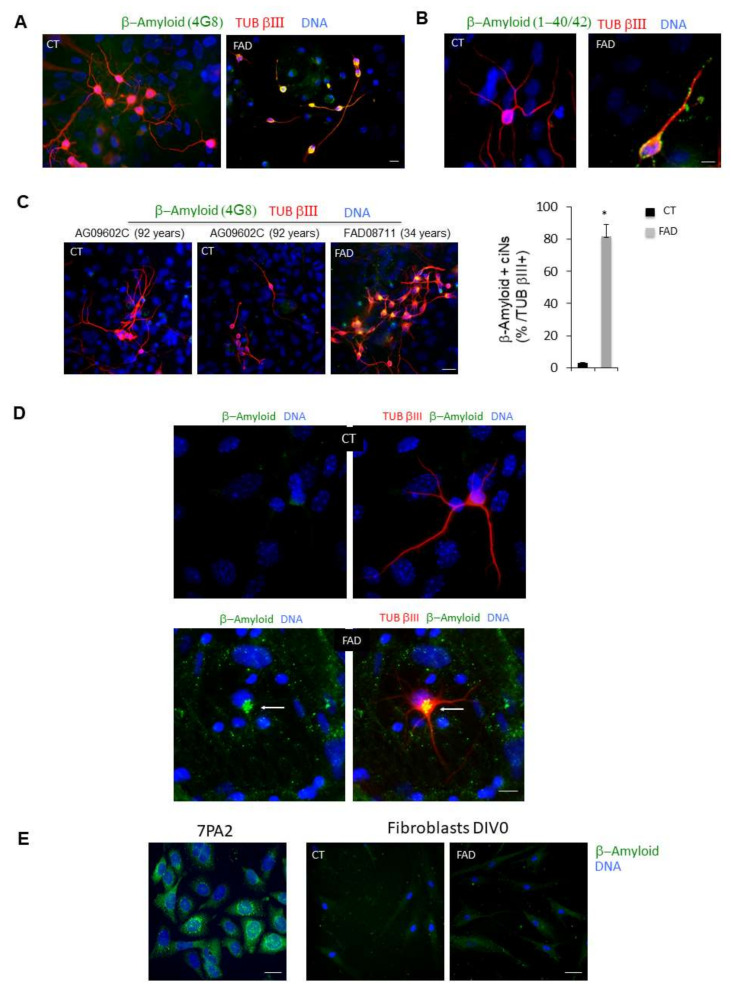
ciNs derived from FAD fibroblasts show intracellular β-Amyloid accumulation. (**A**) Confocal images show that, following chemical induction, fibroblasts acquire a neuronal phenotype (TUB βIII staining, red channel), and ciNs from FAD patients show a strong labelling of β-Amyloid, undetectable in CT ciNs. Nuclei were counterstained with Hoechst (blue channel). Scale Bar, 10 μm. (**B**) Close-up view images of ciNs obtained from CT and FAD converted fibroblasts (TUB βIII, red channel) showing the accumulation of β-Amyloid labelled with the antibody that recognises β-Amyloid 1-40/42 (green channel). Nuclei were counterstained with Hoechst (blue channel). Scale Bar, 10 μm. (**C**) Representative fluorescence images of TUB βIII positive ciNs (red channel) obtained from the direct conversion of fibroblasts from an old, healthy subject (92 years) showing the absence of aggregates of β-Amyloid (green channel), as compared with the FAD subject. Scale Bar, 20 μm. Histogram data represent the percentage of FAD ciNs showing β-Amyloid staining expressed as percentage of total number TUB βIII + cells (82 ± 7% β-Amyloid/ TUB βIII positive cells). Bars in the plots represent means ± SD (n = 3, * *p* < 0.05 vs. control values). (**D**) Triple confocal immunofluorescence images of ciNs showing the presence of β-Amyloid aggregates close to the nucleus in FAD cells. This aggregates have features similar to the so called “aggresomes” (white arrow) generally found in the cytoplasm as protein inclusions. β-Amyloid (green channel), TUB βIII (red channel), DNA (blue channel). No β-Amyloid aggregates are observed in CT cells. Double fluorescence images are also shown (green and blue channel) to better visualize the structure. Scale Bar, 10 μm. (**E**) 7PA2 CHO cells, over-expressing mutated Amyloid Precursor Protein (APP), were used as positive control for the staining with the anti β-Amyloid antibody (green channel). Differently, the same antibody failed to show any specific labelling in dermal fibroblasts at DIV 0 from both healthy subjects and FAD patients (Coriell Institute, Biobank, Camden, NJ, USA and NIH, Bethesda, MD, USA), thus indicating that direct chemical conversion of fibroblasts into neurons is crucial for detection of the pathological marker. Nuclei were counterstained with Hoechst (blue channel). Scale Bar, 20 μm.

**Figure 4:**
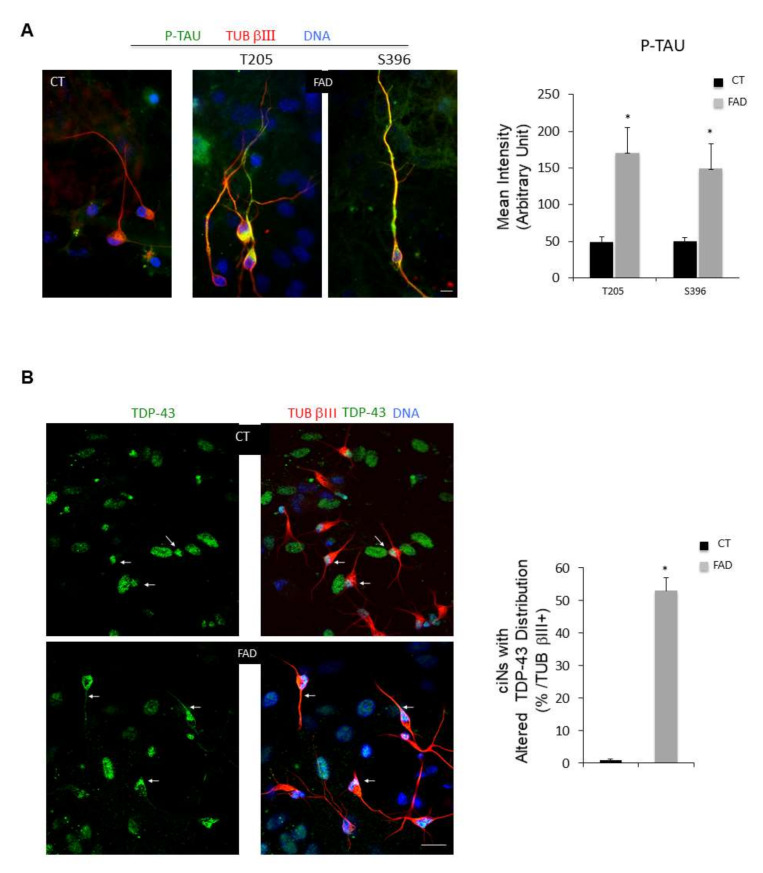
ciNs derived from FAD fibroblasts show an increase in P-TAU immunoreactivity and altered TDP-43 localization. (**A**) Representative triple fluorescence images of ciNs (TUB βIII labelling, red channel), obtained from chemically converted FAD fibroblasts, showing a strong labelling with two different anti P-TAU antibodies (green channel) (Thr205 and Ser396). Nuclei are counterstained with Hoechst (blue channel). Scale Bar, 10 μm. Histogram data represent the mean intensity values (arbitrary unit) of P-TAU immunolabelling in ciNs of CT and FAD by using two different phospho-antibodies (T205 and S396). Bars in the plots represent means ± SD from three independent experiments (* *p* < 0.05) (**B**) Fluorescence images of ciNs derived from CT and FAD chemically converted fibroblasts, triple labelled with TDP-43 (green), TUB βIII (red) and DNA (Blue). White arrows point to TDP-43 labelling distribution. In CT cells, all cells displayed TDP-43 exclusively accumulated in nuclei, whereas in FAD cells, TDP-43 labelling is decreased in the nucleus and redistributed along neurites. Scale Bar, 20 μm. Histogram represents the percentage of FAD ciNs showing altered TDP-43 distribution. Bars in the plots represent means ± SD from three independent experiments (* *p* < 0.05).

**Figure 5:**
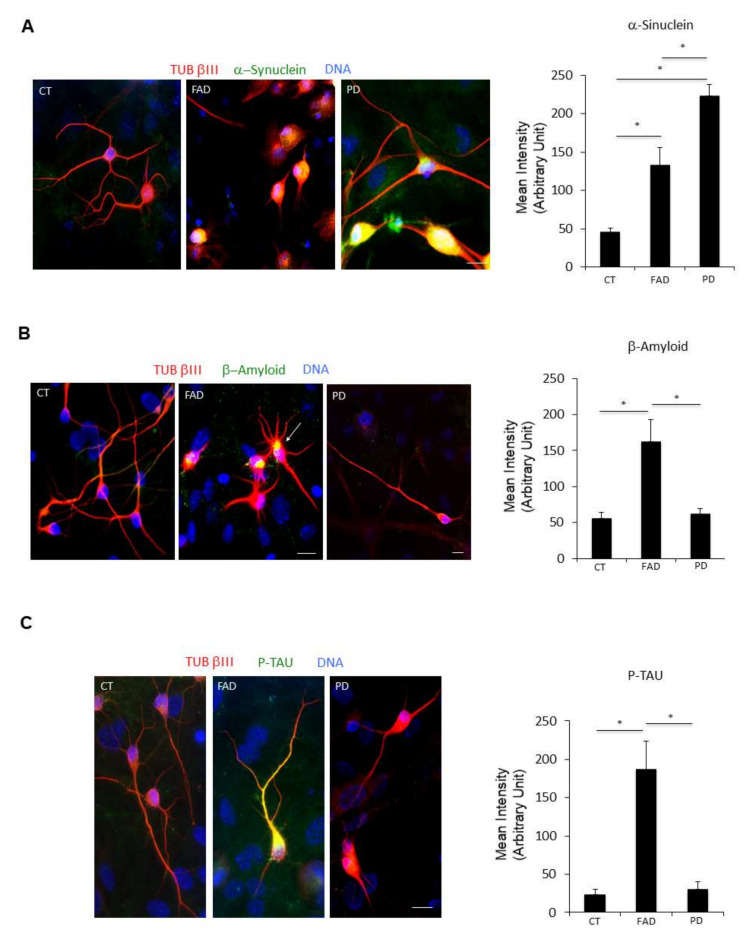
ciNs from FAD and PD patients show neurodegenerative disease hallmarks. (**A**) Immunofluorescence images of ciNs labelled with an anti α-Synuclein antibody (green channel) and the neuronal marker TUB βIII (red channel). Nuclei were counterstained with Hoechst (blue channel). α-Synuclein antibody was able to strongly label PD ciNs, showing aggregates within the cell. Similarly, FAD ciNs show the presence of α-Synuclein labelling and aggregates, although with a weaker intensity. Scale Bar, 10 μm. Histogram data represent the mean intensity values (arbitrary unit) of α-Synuclein immunolabelling in ciNs of CT, FAD and PD. Bars in the plots represent means ± SD from three independent experiments (* *p* < 0.05, one-way ANOVA). (**B**) Immunofluorescence images of ciNs obtained from direct conversions of CT, FAD and PD patient fibroblasts, labelled with an anti β-Amyloid antibody (green channel) and the neuronal marker TUB βIII (red channel). Nuclei were counterstained with Hoechst (blue channel). The images show that FAD ciNs have a strong labelling, with the anti β-Amyloid antibody consisting of aggregates localised close to the nucleus (white arrow), as compared with CT cells. On the contrary, PD ciNs do not show any evident aggregates of β-Amyloid. Scale Bar, 10 μm. Histogram data represent the mean intensity values (arbitrary unit) of β-Amyloid immunolabelling in ciNs of CT, FAD and PD. Bars in the plots represent means ± SD from three independent experiments (* *p* < 0.05, one-way ANOVA). (**C**) Immunofluorescence images of ciNs labelled with an anti P-TAU antibody (Ser396, green channel) and the neuronal marker TUB βIII (red channel). Nuclei were counterstained with Hoechst (blue channel). P-TAU antibody was able to label the cell body and neurites of FAD ciNs, whereas it failed to stain PD ciNs. Scale Bar, 10 μm. Histogram shows the mean intensity values (arbitrary unit) of P-TAU immunolabelling in ciNs of CT, FAD and PD. Bars in the plots represent means ± SD from three independent experiments (* *p* < 0.05, one-way ANOVA).

We then looked at typical pathological markers of neurodegenerative diseases, including AD and PD, which could be easily detected in ciNs using immunofluorescence microscopy. Using two different antibodies specific for β-Amyloid (Figure 3A,B), we found that FAD ciNs, but not ciNs from age-matched control subjects, show a strong β-Amyloid labelling (Figure 3A–D). This staining is not detected even in a very old healthy subject (Figure 3C). In particular, FAD ciNs show the presence of β-Amyloid aggregates close to the nucleus that share features with aggresomes (Figure 3D, white arrow). Indeed, aggresomes are cytoplasmic inclusions localized at the centrosome adjacent to the nucleus and are linked to the pathogenesis of many neurodegenerative diseases [15]. Importantly, β-Amyloid immunoreactivity was not detected in FAD fibroblasts but accumulated following conversion of fibroblasts into neurons (Figure 3E). This result indicates that direct chemical conversion of fibroblasts into neurons is crucial for detection of this pathological marker. Moreover, we found an increase in P-TAU immunoreactivity in ciNs from FAD patients by using two different phospho-antibodies (T205: CT 49.8 ± 6.9, FAD 171 ± 33; S396: CT 50.6 ± 5.1, FAD 149 ± 33.7), distributed in cell body and along neurites (41 ± 7% P-TAU/TUB βIII positive cells) (Figure 4A). A similar percentage of cells from healthy subjects were stained, although with a weaker and more diffuse labelling, limited to the cell body. Stress-mediated regulation of TDP-43 localization, aggregation and neuropathology have been observed in a spectrum of distinct neurodegenerative disorders [16]. Indeed, we observed an altered nucleus/cytoplasm distribution of TDP-43 protein (Figure 4B) rather than a difference in labelling intensity. As shown in Figure 4B, right panel, the percentage of ciNs with altered TDP-43 nucleus/cytoplasm distribution was 53 ± 4% TDP-43/TUB βIII positive cells. Interestingly, we found α-Synuclein aggregates both in FAD and PD patients, with a stronger intensity of labelling in PD ciNs (Figure 5A and Figure S2). On the contrary, ciNs from PD patients did not show either β-Amyloid accumulation or P-TAU immunoreactivity (Figure 5B,C). Histogram data in Figure 5 represent the mean intensity of pathological markers immunolabelling in ciNs of CT, FAD and PD: α-Synuclein (CT 46 ± 5, FAD 133 ± 23, PD 224 ± 14), β-Amyloid (CT 56 ± 8.1, FAD 163 ± 30, PD 62 ± 7.3) and P-TAU (CT 24 ± 6.3, FAD 187 ± 36.2, PD 31 ± 9.2). Therefore, ciNs not only retain important aspects of AD and PD pathology but, in our specific analysis, could also allow for a differential diagnosis. This is clinically relevant, since the symptoms of different neurodegenerative diseases have a high degree of similarity.

### 2.3. Detection of Mitochondrial Abnormalities in Patient ciNs

**Figure 6:**
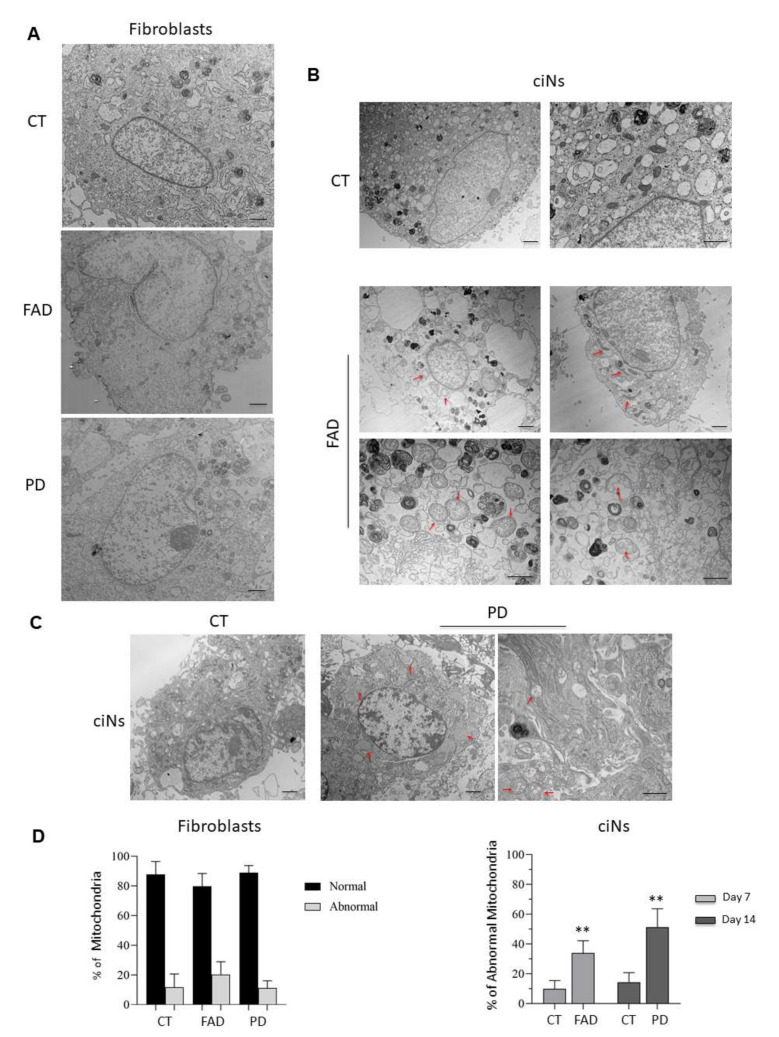
Ultrastructural analysis and morphometric quantification of abnormal mitochondria. (**A**) Representative TEM images of healthy (CT), FAD and PD fibroblasts. Mitochondria with normal morphology appear with round shape and densely packed cristae in all three experimental groups. Scale Bar, 1 μm. (**B**) Morphometric analysis of TEM images at a 2500× (Scale Bar, 1 μm) and 6000× magnification (Scale bar 800 nm) of ciNs from CT and FAD patients. Images show mitochondrial abnormalities (red arrows) mainly consisting of elongated and distorted shape and swollen with a less dense matrix. (**C**) Morphometric analysis of TEM images at a 2500× (Scale Bar, 1 μm) and 6000× magnification (Scale bar 800 nm) of ciNs from CT and PD patients. Images show mitochondria swollen with a less dense matrix, and swollen with severe loss of cristae with an empty matrix (red arrows). (**D**) Quantitative assessment of *mitochondrial* morphology. The number of normal and abnormal mitochondria was measured in both fibroblasts and ciNs of CT, FAD and PD patients in 12 different fields of view (190 μm^2^). Percentage of abnormal mitochondria in ciNs of FAD and PD patients was calculated on the total number of counted mitochondria (approx. n = 500/experimental group). Data are depicted as mean ± SD, n = 3 independent cell cultures. ** *p* < 0.01 (two-way ANOVA).

The identification of early biomarkers for neurodegenerative diseases may allow the development of early, personalised and rehabilitative interventions. There is strong evidence that mitochondrial dysfunction and abnormal mitochondrial dynamics occur early, thus potentially representing early biomarkers of neurodegeneration [17,18]. To investigate whether mitochondria of ciNs derived from patient fibroblasts show ultrastructural abnormalities, as compared with healthy subjects, we carried out transmission electron microscopy (TEM) analysis. Mitochondrial evaluation was also performed in fibroblasts of the different groups to detect possible differences in starting conditions. We found that normal mitochondria were round in shape, containing packed cristae and dense matrix in fibroblasts of both healthy subjects and patients (Figure 6A). Although some ultrastructural abnormalities in mitochondrial shape were occasionally observed, there were no significant differences among the groups (Figure 6D, left panel). On the contrary, quantitative morphometric studies of ciNs from patients showed the following mitochondrial abnormalities: elongated, elongated and distorted, swollen with less dense matrix, and swollen with severe loss of cristae with an empty matrix (Figure 6B,C and Table S1). In particular, FAD ciNs demonstrated approximately 34 ± 8% damaged mitochondria, consisting of enlarged and elongated mitochondrial forms (Figure 6B,D right panel). In PD ciNs, mitochondria were morphologically large, swollen and with reduced crests (51 ± 12%) (Figure 6C,D right panel). Similar ultrastructural abnormalities have been reported in cellular and animal models of neurodegeneration as consequences of mitochondrial fission/fusion and mitochondrial dysfunction [19,20].

### 2.4. Reprogramming of Human Fibroblats on Microfluidic Devises and Detection of Neuropathological Markers

**Figure 7:**
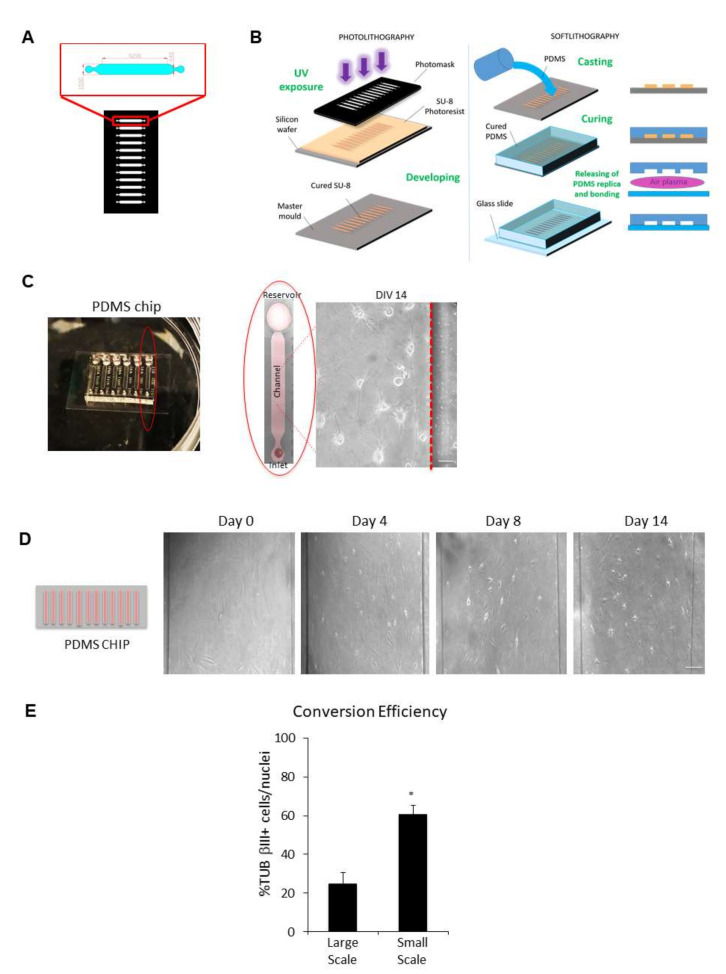
Microfluidic technology combined with chemical reprogramming of fibroblasts enable neuronal differentiation. (**A**) Microfluidic channel design: the high resolution photomask is a glass layer directly printed from a CAD file by a laser-writer. Microchannel areas are transparent, while complementary area is black and opaque. The photomask includes a series of microchannels with the shape shown in the red zoom box (dimension in micrometres). (**B**) Photolithography (left panel): the SU-8 negative photoresist layer is spun with a 200 μm of thickness on a silicon wafer and prepared with a pre-baking (15 min at 95 °C) for UV light etching. After light exposure through the photomask, crosslinked areas with the microchannel shape are obtained on the photoresist layer. A post-baking (7 min at 95 °C) treatment and a final chemical bath (developing) remove the uncrosslinked areas, and after a supplementary hard baking, the final mould is obtained. Soft lithography (right panel): the replica of the mould is obtained through a casting process of liquid 1:10 PDMS (Polydimethylsiloxane). The transparent polymer is thermally cured (30 min at 80 °C) and removed from the mould. A plasma treatment is used to bond the PDMS structure to a glass slide. (**C**) Representative image of the chip device used in this study. A single channel is outlined by a red ellipse. Cells were chemically converted directly into the channel, as shown in the phase-contrast image of ciNs at DIV 14. Scale Bar, 10 μm. (**D**) (Left panel) A schematic drawing of a typical chip device with 12 microchannels in which cells are directly converted. (Right panel) Phase-contrast images showing the direct conversion of human fibroblasts from a healthy subject on chip with small molecules. The time-course of chemical conversion starts from fibroblasts (DIV 0) with the typical flat and elongated morphology. Soon after the addition of CA medium, followed by CB medium addition (DIV 1 up to DIV 14), an increasing number of fibroblasts on chip change their morphology and acquire small rounded nuclei with cytoplasmic protrusions. Particularly, morphological changes appear slowly in the first days of chemical conversion and then enhance during DIV 4 up to DIV 8. Scale Bar, 50 μm. (**E**) Histogram showing a comparison between the efficiency of transdifferentiation in large-scale (24-well plate) and in small-scale (chip) chemical conversion procedures. Data are shown as percentage of TUB βIII positive cells/nuclei. A strong increase in TUB βIII positive cells with a neuronal morphology is observed upon chemical conversion directly on chip. Bars in the plots represent means ± SD (n = 3, * *p* < 0.05).

So far, induced neurons obtained by direct reprogramming of adult somatic cells have been grown and examined using conventional cell culture dishes (large scale) (Figure 1A). The increasing burden of neurological disorders reported worldwide, such as AD and PD, is pushing forward microfluidic and microscale technology application to study the brain and develop microscale assays for multiple cell group analysis, with the added benefit of minimised reagent quantities [21]. In light of this consideration, we focused on the downscaling of direct reprogramming by culturing cells on microfluidic chips (small scale) (Figure 7) made of polydimethylsiloxane (PDMS) scaffolds, which have different properties, such as optical transparency, gas permeability, inert material and biocompatibility (Figure 7A–C). Our PDMS chips contain six cell culture chambers that are 200 μm high, 1.5 mm wide and 9.2 mm long (Figure 7A,C).

**Figure 8:**
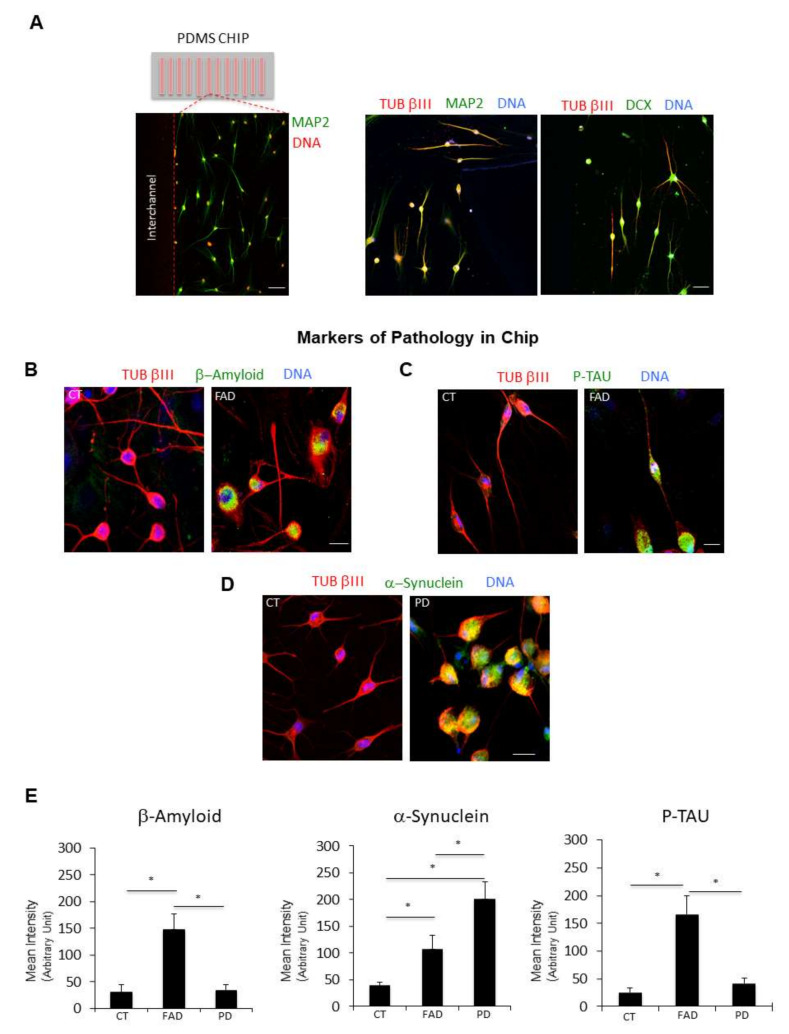
Microfluidic technology combined with chemical reprogramming of fibroblasts enable detection of neuropathological markers. (**A**) (Left panel) A low magnification confocal immunofluorescence image shows the differentiation of cells expressing the late neuronal marker MAP2 (green channel). Nuclei are counterstained with Hoechst (red channel). (Right panel) Two representative confocal immunofluorescence images at higher magnification show ciNs expressing the neuronal markers TUB βIII, MAP2 and DCX. Scale Bar 20 μm. (**B**) Close-up view of ciNs, directly converted on chip, from CT and FAD patients, labelled with β-Amyloid antibody (green channel) and the neuronal marker TUB βIII (red channel). Nuclei were counterstained with Hoechst (blue channel). ciNs from FAD fibroblasts show accumulation of the pathological marker β-Amyloid. Scale Bar, 10 μm. (**C**) Close-up view of ciNs, directly converted on chip, from CT and FAD patients, labelled with P-TAU (Thr205) antibody (green channel) and the neuronal marker TUB βIII (red channel). Nuclei were counterstained with Hoechst (blue channel). ciNs from FAD fibroblasts show a strong labelling with the anti-P-TAU antibody. Scale Bar, 10 μm. (**D**) ciNs of CT and PD subjects were labelled with α-Synuclein antibody (green channel) and the neuronal marker TUB βIII (red channel). Nuclei were counterstained with Hoechst (blue channel). Chemically converted cells on chip confirm the presence of α-Synuclein aggregates only in patient cells as compared with CT cells. Scale Bar, 10 μm. (**E**) Histogram data represent the mean intensity values (arbitrary unit) of β-Amyloid, α-Synuclein and P-TAU immunolabelling in ciNs of CT, FAD and PD. Bars in the plots represent means ± SD from three independent experiments (* *p* < 0.05, one-way ANOVA).

We found that the application of chemical transdifferentiation to microfluidic devices allows the generation of neuronal cells directly on microchannels (Figure 7C,D) with a higher transdifferentiation efficiency (61 ± 4.3% of TUB βIII positive cells/nuclei), as compared with ciNs on 24-well plates (transdifferentiation efficiency 25 ± 5.4% of TUB βIII positive cells/nuclei) (Figure 7E). The ciNs on chip express typical neuronal markers, including TUB βIII, DCX and the late neuronal marker MAP2 (Figure 8A). Most importantly, we show that ciNs on chip from FAD fibroblasts, but not from healthy fibroblasts, express pathological markers, such as β-Amyloid (Figure 8B,E; mean intensity: CT 31 ± 14, FAD 148 ± 29, PD 33 ± 11.5) and P-TAU (Figure 8C,E; mean intensity CT 25 ± 8, FAD 166 ± 34, PD 42 ± 9.5). In addition, PD ciNs cultured on chip, show the presence of a high level of α-Synuclein immunoreactivity (Figure 8D,E; mean intensity: CT 39 ± 6.2, FAD 107 ± 24.7, PD 202 ± 30.2). These results confirmed those obtained in large-scale experiments.

## 3. Discussion

This study describes for the first time the development of a novel, scalable, non-invasive and cost-effective cell-based tool that can prove useful for the diagnosis of protein misfolding neurodegenerative diseases.

The main findings are that (i) patient fibroblasts have a reduced efficiency of reprogramming, and chemically reprogrammed cultures present a less complex neuronal network; (ii) ciNs from patients show misfolded protein accumulation, a neurodegenerative disease hallmark; (iii) patient ciNs show mitochondria ultrastructural abnormalities, an early marker of neurodegeneration; (iv) microfluidic chip technology, coupled with chemical reprogramming of adult fibroblasts, enables on-chip examination of disease pathological processes and may have important applications in high-throughput diagnosis.

Nowadays, β-Amyloid, α-Synuclein and P-TAU are considered core candidate biomarkers for the certainty of clinical diagnosis of AD and PD. Here, we describe a novel cellular approach for neurodegenerative disease diagnosis by detection of these neuropathological markers, easily revealed by conventional immunofluorescence. Indeed, we were able to show that FAD ciNs have β-Amyloid aggregates distributed close to the nucleus, similar to aggresomes, which are generally described in neurons over-expressing misfolded proteins [15]. However, whether the presence of aggregates in patient neurons is a cause or a consequence of the cellular pathology has yet to be convincingly established. Thus, further studies carried out using ciNs might be important to elucidate the underlying cellular machinery involved in aggresome formation and function.

Moreover, in accordance with Mertens et al. (2021) [8], we found that FAD ciNs show an altered morphology consisting of reduced neurite length and network, probably due to FAD cell inability to reach a mature state during reprogramming. Differently from Mertens and colleagues, we observed a reduced reprograming efficiency of FAD fibroblasts. This can be due to several factors, the most important of which ais lentivirus-mediated forced expression of specific transcription factors (Ngn2 and Ascl1), which might override endogenous differences.

Therefore, the direct chemical reprogramming-based cell model may represent a promising scaffold for phenotypic analysis and thereby be representative as a patient-specific “disease-in-a-dish” model. It is noteworthy to mention that immunolabelling approaches, detecting different pathological markers, are routinely utilised for the diagnosis of cancer in patient biopsies, and thus their application in neurodegenerative disease diagnosis might be also envisaged. Overall, the proposed method may have important clinical implications in simplifying patient characterization along with other imaging and neurological analysis commonly used for diagnosis and staging the pathology.

Although it is possible to foresee an innovative clinical application of the chemical reprogramming approach, its main disadvantages consist of producing a reduced number of neurons and a mixed population of cells with different degrees of maturity and a unique subtype of neurons, which are generally excitatory though capable of maintaining the age-related features associated with human pathology. However, the resulting neuronal heterogeneity at differentiation levels may resemble the endogenous condition of the patient brain, even though suitable for single cell analysis, including immunofluorescence, electrophysiology and transcriptomics.

Moreover, considering that in large-scale experiments we use a mouse glial monolayer to induce neuronal maturation, it could be envisaged to test the effects on ciNs of glia cells obtained from healthy or patient Neural Progenitor Cell (NPC) subjects and analyse cell-autonomous or non-autonomous phenotypes.

Mitochondrial dysfunction is a central hallmark of nearly all neurodegenerative diseases, playing a quite upstream role in the cascade of pathological events that link bioenergetics and misfolded protein toxicity [22]. Neurons are highly dependent on mitochondrial function; because of their high energy demands, they represent the most suitable system to investigate the appearance and role of mitochondrial alterations. The majority of observations about mitochondrial abnormalities in neurodegeneration come from studies on animal models and post-mortem analysis of the patient’s brain [18,23,24]; hence, our ultrastructural analysis conducted in patient-derived neurons, available after a short time in culture, represents an exclusively important early source of information for neurodegenerative disease diagnosis and studies. Mitochondrial dysfunction has recently been detected in cells other than brain cells, such as blood cells and fibroblasts from AD patients [17,22,25,26,27]. However, these data are controversial,, and studies on fibroblasts have reached little consensus [26,27]. Indeed, although both sporadic AD and FAD patient fibroblasts showed some mitochondrial abnormalities, the level of defect varies, and only a subset of fibroblasts analysed presented mitochondrial dysfunction. Although we failed to detect significant ultrastructural abnormalities in mitochondria of patient fibroblasts, whose integrity is essential for neuronal differentiation [28,29], FAD fibroblasts’ reduced efficiency of direct conversion may be caused by their higher mitochondrial vulnerability to the stressful condition of reprogramming [8], as compared with healthy cells. Indeed, the rapid metabolic transition that takes place during the fate switch from fibroblast to neuron puts enormous stress on the cell, leading to the formation of ROS; thus, in FAD cells, which have a higher baseline of ROS-induced stress condition [8], this may represent the major barrier to transdifferentiation and justify the reduced efficiency of neuronal reprogramming.

Pathological cellular phenotypic changes, such as misfolded protein accumulation, often manifest several years before the initial onset of symptoms and are driven by early pathogenic processes in the so-called “prodromal” phase [30]. It is thus reasonable to hypothesise that the phenotypic changes observed in our patient-specific “disease in a dish” model, including altered neuronal morphology and mitochondrial ultrastructural alterations, might be exploited as early neurodegenerative signals, thus offering an important window of diagnosis for more precocious interventions. Moreover, our study can be considered as a possible starting point for clinician reference because provides critical information to distinguish between the two neurodegenerative disorders analysed: FAD and PD. Therefore, this model could be not only a general predictor of neurodegenerative disorders but also a potential tool to discriminate between these two major diseases. Despite the limited number of patients, the results obtained are unambiguous because healthy subject cells do not display significant pathological alterations, and the extent of the absolute difference between cases and controls supports our conclusions. However, further studies with a large number of patients, including mildly cognitive-impaired patients, the sporadic form of AD and risk-associated variants, will be necessary to validate this approach as a routine tool for diagnosis.

The combination of direct chemical reprogramming-based cell models with microfluidic technology enables up to hundreds of experiments to be performed in parallel by a single operator, and this number can be further implemented by coupling microfluidic technology with automatic liquid handlers. Moreover, the setup of cultures on microfluidic devices represents an important implementation in our approach, since it leads to a remarkable increase in cellular reprogramming efficiency, experimental throughput— which is critical for population studies—and a valid alternative to other in vitro cellular models. In this respect, human-induced pluripotent stem cells (iPSCs) require a long time to differentiate into functional neurons (months), and the resetting of epigenetic information during reprogramming may miss the information imparted by age. On the contrary, direct chemical conversion of somatic cells into neuronal cells allows the preservation of the signatures of donors’ age [31].

In conclusion, our study shows that chemically reprogrammed cultures on chip represent a rapid and convenient system to detect neurodegenerative disease hallmarks, thus providing an experimental platform for the study of pathogenic mechanisms, biomarker discovery and design of personalised therapeutic strategies.

## 4. Materials and Methods

### 4.1. Study Design and Chemical Reprogramming

In this study, we performed both large-scale and low-scale direct conversion experiments using fibroblasts with known phenotypes from Coriell Institute Biobank, Camden, NJ, USA, including healthy subjects, patients affected by Familiar Alzheimer’s Disease (FAD/PSEN1) and patients affected by Parkinson’s Disease (PD).

#### 4.1.1. Cell Culture

Human fibroblasts were maintained in DMEM (Life Technologies, Monza, Italy) supplemented with 10% foetal bovine serum (Life technologies, Monza, Italy), 1 mM GlutaMAX (Life Technologies, Monza, Italy), 0.1 mM non-essential amino acid (NEAA, Millipore, Burlington, MA, USA) and penicillin/streptomycin (Life Technologies, Monza, Italy) at 37 °C with 5% CO_2_.

Primary skin fibroblasts from healthy subjects and patients were obtained from Coriell Institute for Medical Research, Camden, NJ, USA. See Table 1 for details about age, sex and disease of the correspondent patient whose cells were used. The use of fibroblasts was restricted to a maximum of five cell passages to avoid replicative senescence, and cultures were always maintained below confluence.

Primary astrocytes were isolated from the brain of newborn mice at 1 day post-birth [32]. Animal experiments were performed in accordance with relevant institutional and national regulations (protocol nr.90/2016-PR).

Briefly, mouse pups were sprayed with 70% ethanol and sacrificed by decapitation. The brain was placed in a dissecting dish filled with cold D-HANKS medium, and the dissection procedures were performed under a stereomicroscope. Olfactory bulbs, cerebellum and meninges were carefully removed using dissecting forceps. The cortical cerebral hemispheres were transferred into a Falcon tube and digested with 1.25% trypsin and DNAse (Sigma-Aldrich, Milan, Italy) in a water bath at 37 °C for 15 min. After washes in MEM containing 1 mL 30% D-glucose, 10% foetal bovine serum and penicillin/streptomycin, 10 mL of this medium was vigorously pipetted to dissociate tissue pieces into single cells. Cells were then counted and plated in a T75 culture flask and incubated for at least 9 days at 37 °C in the CO_2_ incubator. Glial cells were cultured for at least 3 passages in T175 flasks. Before detachment with trypsin, the cultures were thoroughly washed with PBS to minimize contamination by overlaying microglia and oligodendrocytes.

Glia cells at passage 3–4 were then plated in 24-well plates containing glass coverslips (12 mm, Menzel-Glaser, Thermo Fisher Scientific, Waltham, MA, USA), previously coated with Matrigel (Corning) diluted in DMEM/F12 (Life Technologies, Monza, Italy) (1:40). One day after plating, the medium was changed, and the astrocyte cultures were maintained in DMEM (Aurogene, Rome, Italy) supplemented with 10% foetal bovine serum (Life Technologies, Monza, Italy) and penicillin/streptomycin. Generally, 200,000 glial cells were plated to generate a uniform monolayer to be used within 3 days for seeding of chemically induced fibroblasts.

A Chinese hamster ovary (CHO) cell line over-expressing the Val717Phe FAD mutation in the APP gene that leads to β-Amyloid 1–42 overexpression, 7PA2, was a kind gift from Prof. d’Erme’s laboratory (Sapienza University, Rome, Italy) [19]. The cell line was maintained in DMEM/F12 supplemented with 10% FBS, 10 M glutamine, penicillin/streptomycin 1X and 200 μg/mL G418 for transgene selection at 37 °C and in a CO_2_ humidified incubator. Cells were grown on coverslips for immunofluorescence analysis and used as control for labelling with anti β-Amyloid antibodies.

#### 4.1.2. Large-Scale Experiments on Coverslips

Twenty-four-well plates with or without coverslips were coated with 0.1% gelatin (Sigma-Aldrich, Milan, Italy) for at least 30 min at 37 °C. Initial fibroblasts were seeded onto gelatin-coated culture plates (15,000 cells/well) and cultured in fibroblast medium for 24 h. After one day, the sub-confluent cells were exposed to the neuronal induction medium (DMEM/F12: Neurobasal (1:1), 0.5% N-2, 1% B-27, 100 μM cAMP, 20 ng/mL βFGF) with the chemical cocktail VCRFSGY, as reported by Hu et al. (2015) [11]. The concentration of the chemicals used was VPA, 0.5 μM; CHIR99021, 3 μM; Repsox, 1 μM; Forskolin, 10 μM; SP600125, 10 μM; GO6983, 5 μM; Y-27632, 5 μM. The same medium was replaced after 3 days. After 7 days, chemically induced cells were detached by 5 min exposure to Tryple (Life Technologies, Monza, Italy) (70 μL/well) and re-plated on a layer of glia, previously obtained from newborn mice. Induced cells from 3 wells were generally pulled and seeded on glia cells in neuronal maturation medium consisting of DMEM/F12: Neurobasal (1:1), 0.5% N-2, 1% B-27, 100 μM cAMP, 20 ng/mL BDNF, 20 ng/mL GDNF, 20 ng/mL NT3 and penicillin/streptomycin with CFD (CHIR99021, 3 μM; Forskolin, 10 μM; and Dorsomorphin, 1 μM). Half of the maturation medium was replaced with new medium every other day. At 14 days in culture, cells were kept in DMEM/F12: Neurobasal (1:1), 0.5% N-2, 1% B-27, 20 ng/mL BDNF, 20 ng/mL GDNF, 20 ng/mL NT3 and penicillin/streptomycin and then fixed for immunofluorescence analysis (DIV17). The morphology of the differentiating cells in different induction groups was analysed at various time points by phase contrast microscopy (DMIL microscopy, Leica, Wetzlar, Germany) and the images acquired using a photocamera DFC420 C (Leica).

#### 4.1.3. Small-Scale Experiments on Chip

The procedure followed the protocol by Yang et al. (2019) [12], with some modifications and adaptation for the chip technology.

Human fibroblasts were first cultured on Matrigel-coated chips at 80 cells/mm^2^. The initial cell density on chip is very important for efficiency of neuronal induction and differentiation. After seeding on chip, cells were maintained in fibroblast medium for 24 h. The medium was changed twice a day (10 μL/channel) for 14 days. Next, the medium was completely replaced by neuronal induction medium (NIM) consisting of DMEM/F12 (1:1) supplemented with 1% N-2, 2% B-27, 40 ng/mL BDNF, 20 ng/mL GDNF, 20 ng/mL insulin-like growth factor 1, 1 mM GlutaMAX and 100 μM cAMP. Cells were treated with NIM containing CA (3 μM CHIR99021, 0.25 μM LDN193189, 10 μM RG108, 1 μM Dorsomorphin, 3 μM P7C3-A20, 0.5 μM A83-01, and 5 μM ISX9). Three days later, the medium was changed to NIM containing CB (10 μM forskolin, 5 μM Y27632, 1 μM DAPT, 1 μM PD0325901, 0.5 μM A83-01, 1 μM purmorphamine, and 3 μM P7C3-A20) for another 3 days. From day 12, half of the medium was replaced with neuronal maturation medium (NMM): NIM supplemented with 20 ng/mL NT-3 and 2 μM Y27632 every 2 days. Changes in the morphology of differentiating cells in different induction groups were observed at various time points by phase contrast microscopy (DMIL microscopy, Leica) and images acquired using a photocamera DFC420 C (Leica). At day 14, cells on chips were fixed and analysed as described below.

### 4.2. Chemicals

Small molecular compounds were dissolved and diluted in dimethyl sulfoxide (Sigma-Aldrich, Milan, Italy) or sterile ultrapure water (Gibco, Thermo Fisher Scientific, Waltham, MA, USA) and then processed to use at the final concentrations. All small molecular compounds were purchased and dissolved as recommended by the company (Table S2). Growth factors were purchased from Peprotech and diluted according to the manufacturer’s instructions.

### 4.3. Chip

Microfluidic platforms were fabricated according to standard soft-lithographic techniques and moulded in polydimethylsiloxane (PDMS), as previously described [9]. The microfluidic chip consists of a series of 6 independent parallel channels, each endowed with an entry and exit port for connection to the external microfluidic applied by pipetting. The chip consists of a PDMS layer fabricated by replica moulding and bonded to a glass slide by plasma treatment. The main steps of the fabrication procedure consist of a photolithography phase followed by a soft-lithography phase. A high resolution photomask is printed on a glass layer starting from a CAD (Computer Aided Design) file. Following standard soft-lithography techniques, a 200 μm thick layer of SU-8 (negative) photoresist is spun over a silicon wafer and is successively prepared for etching with UV light by pre-baking (15 min at 95 °C). Light exposure through the photomask produces the desired pattern on the photoresist layer by crosslinking the exposed areas. After a post-baking (7 min at 95 °C) treatment and a final chemical bath (developing) to remove the excess material, the final mould is eventually obtained by a supplementary hard baking. After polymerization, the PDMS chip was cut from the mould, and both ends of each channel were punched to realize the “inlet” injection site and the “reservoir”. Later, the chip was sealed on a 60 × 24 mm microscope cover glass slide (Menzel-Glaser, Thermo Fisher Scientific, Waltham, MA, USA) by plasma activation (Harrick Plasma Cleaner). Channels were rinsed twice with isopropanol and distilled water to check proper flow before autoclaving.

### 4.4. Immunofluorescence on Coverslip

Immunofluorescence was performed as previously described [33]. Cells were fixed for 10 min in 4% paraformaldehyde in PBS containing 4% sucrose, permeabilised with 0.2% Triton X-100 in PBS for 3 min and washed three times for 5 min with PBS. All antibodies were diluted in PBS containing 3% bovine serum albumin and 0.05% Tween-20 and incubated for 1 h at 37 °C. The following primary antibodies were used (60 min, 37 °C): TUB βIII (1:500, MAB1637 Rb, Millipore, Burlington, MA, USA and NB110-57611 mouse, Novus Biologicals, Littleton, CO, USA), TAU (1:200, MAB10417 rabbit, Millipore, Burlington, MA, USA), MAP2 (1:250, AB5622 rabbit, Millipore, Burlington, MA, USA) and (M4403 mouse, Sigma-Aldrich, Milan, Italy), NeuN (1:250, ABN78 rabbit, Millipore, Burlington, MA, USA), Synapsin I (1:250, AB1543 Rb, Millipore, Burlington, MA, USA), DCX (1:300, sc-8066 goat, Santa Cruz), β-Amyloid 4G8 (1:200, SIG39220 mouse, Millipore, Burlington, MA, USA), β-Amyloid 1-40/42 (1:200, AB5076 rabbit, Millipore, Burlington, MA, USA), GFAP (1:500, AB5804 rabbit, Sigma-Aldrich, Milan, Italy), α-Synuclein (1:250, sc-12767 mouse, Santa Cruz, Dallas, TX, USA), phospho-TAU T205 (1:200, ab4841 rabbit, Abcam, Cambridge, UK), phospho-TAU S396 (1:200, sc-101815 rabbit, Santa Cruz, Dallas, TX, USA), TDP43 (1:200, 6H6E12 mouse, Proteintech). After primary antibody incubation and rinsing, the coverslips were incubated with fluorescently labelled secondary antibodies: anti-mouse, anti-rabbit or anti-goat IgGs alexa-488, -555 or -647 (Molecular Probes, Millipore, Burlington, MA, USA), which were used in different combinations according to the primary antibodies. Nuclei were counterstained with Hoechst 33258 or with propidiumiodide after RNAse treatment, when DNA was shown in the red channel. Coverslips were mounted in Prolong Glass antifade mountant, and samples were first observed with an Eclipse 80i Nikon Fluorescence Microscope, equipped with a VideoConfocal (ViCo) system. Final images were also acquired with a Laser Scanning Confocal Microscope (Olympus FluoView FV1000, Olympus Inc.).

For immunolabelling intensity analysis, the quantification was done by measuring the fluorescence using the ImageJ software (NIH, Bethesda, MD, USA, https://imagej.nih.gov/ij, downloaded on 7 June 2016) and represented as plotting histograms of the mean intensity and standard deviation (SD). Images of transdifferentiated cells (n = 10) were acquired from at least three randomly selected fields in three independent experiments. For each cell, we first defined the background level by drawing a 20 μm diameter circle near the soma, in order to define the average fluorescence in the circle as background fluorescence. Then, we drew a circle along the soma and measured the average fluorescence inside and subtracted the previously calculated background value. The resulting value was considered the specific fluorescence label and expressed as mean intensity values with an arbitrary unit. Data were plotted as average and standard deviation.

The efficiency of transdifferentiation was calculated using the cell count plugin from the ImageJ software (NIH, Bethesda, MD, USA, https://imagej.nih.gov/ij, downloaded on 7 June 2016). Percentage efficiency was calculated as the number of TUB βIII positive cells over total Hoechst positive cells (excluding glial cells distinguishable by a very large nucleus) in each visual field, multiplied by 100. Cells were analysed in randomly selected ×20 fields from triplicate samples.

For neurite analysis of ciNs, acquired images were imported into NIH ImageJ, and dendritic complexity was analysed from 8-bit images using the NeuronJ Analysis plug-in. Neurite length represents the length (in pixels) of the longest path from a neuron body to an extreme segment. Total dendritic length of each individual neuron (n = 10) was analysed in triplicate experiments.

### 4.5. Immunofluorescence on Chip

Each channel was rinsed 5 min in Washing Buffer (PBS, Triton X-100 0.1%) three times and incubated for 30 min in Blocking Buffer (PBS, Triton X-100 0.2%, BSA 1%) at room temperature. Primary antibodies were diluted 1:20 to 1:40 in Antibody Solution (DPBS, Triton X-100 0,1%, BSA 1%). Chips were incubated overnight at 4 °C. Next, channels were rinsed again for 5 min in Washing Buffer three times and incubated 1 h at room temperature with fluorescently labelled secondary antibodies: anti-mouse, anti-rabbit or anti-goat IgGs alexa-488, -555 or -647 (Molecular Probes, Millipore, Burlington, MA, USA), which were used in different combinations according to the primaries used for labelling. All the secondary antibodies were diluted 1:250 in Antibody Solution containing Hoechst33258 1:1000. Then, channels were rinsed again 3 times and left in PBS. Images from chips were acquired with a confocal Nikon Eclipse Ti2 microscope.

### 4.6. Electron Microscopy

Biological samples were collected and fixed overnight in 2% glutaraldehyde with 1% tannic acid in 0.1 M sodium cacodylate, pH 7.4. Samples were rinsed 6 times in the sodium cacodylate buffer and then incubated in 2% osmium tetroxide in the same buffer for 2 h at room temperature and processed following a standard schedule for embedding in Epon resin. Following polymerization overnight at 65 °C, 80 nm sections were cut on a Reighert-Jung Ultra cut E ultramicrotome (Leica Microsystems) and picked up on copper grids to be analysed in a TEM-1400 Plus.

For mitochondria analysis, 12 micrographs were taken for each experimental group. In each field of view (190 μm^2^), mitochondria number and morphology were determined. Quantitative changes in size (length and diameter) were measured using NIH ImageJ. Percentage of total abnormal mitochondria was calculated on the total number of counted mitochondria (approx. n = 500/experimental group). The different altered mitochondria phenotypes were calculated as percentage of the total abnormal mitochondria in FAD and PD ciNs. All micrographs were analysed in a random order and were blinded for phenotype.

### 4.7. Statistics

Comparison between two or three groups was performed using the unpaired two-tailed Student’s *t* test or one-way or two-way ANOVA post hoc tests, respectively. Data distribution was evaluated with normal probability plot and data were analysed using GraphPad. Degrees of statistical significance are presented as * *p* < 0.05 or ** *p* < 0.001. Data in the text and figures are expressed as means ± SD.

Post hoc power calculations were carried out using the software G*Power, version 3.1.9.4, considering an alpha error of 5% with two tails, which was 0.99.

## Figures and Tables

**Table 1:** Human dermal fibroblasts used in the study.

	Fibroblasts	Origin
**Healthy**	AG11732	Female 17 years
	AG04062A	Male 31 years
**Subject**	AG04060	Male 44 years
**(CT)**	AG04148	Male 56 years
	AG07141	Female 66 years
	AG07801	Male 70 years
	AG09602C	Female 92 years
**Familiar**		
**Alzheimer’s**	AG08711	Female 34 years
**Disease**	AG06848	Male 56 years
**(FAD)**	AG06840	Female 56 years
**(A246E)**		
**in PSEN1**		
**Parkinson’s**	AG20442	Male 53 years
**Disease**	AG20446	Male 57 years
**(PD)**	AG20443	Male 71 years
**Idiopathic**	AG08395B	Female 85 years

Both large scale and small scale direct conversion experiments for neuronal and pathological marker detection were carried out by using fibroblasts with known phenotypes, from Coriell Institute Biobank, Camden, NJ, USA, including healthy subjects, patients affected by Familiar Alzheimer’s Disease (FAD/PSEN1) and patients affected by Parkinson’s Disease (PD).

## Data Availability

Data that support the findings of this study are available upon reasonable request and in Supplementary Information.

## Supplementary Materials

The following supporting information can be downloaded at: https://www.mdpi.com/article/10.3390/ijms23042147/s1.

## Abbreviations

AD = Alzheimer’s disease; Ascl1 = Achaete-scute homolog 1; BDNF = Brain-Derived Neurotrophic Factor; cAMP = cyclic adenosine monophosphate; CSF = Cerebrospinal fluid; ciNs **=** chemical induced neurons; iNs = induced neurons; DCX = Doublecortin; FAD = Familial Alzheimer’s Disease; GDNF = glial-derived neurotrophic factor; GFAP = Glial Fibrillary Acidic Protein; MAP2 = Microtubule-associated protein 2; Ngn2 = Neurogenin 2; NeuN = Neuronal Nuclear Protein; NT-3 = Neurotrophin-3; NIM = neuronal induction medium; PD = Parkinson’s disease; PDMS = polydimethylsiloxane; PNS1 = Presenilin 1; P-TAU= Phospho-TAU; TDP43 = TAR DNA-binding protein 43; TEM = transmission electron microscopy; TUB βIII = Tubulin βIII; VPA = Valproic Acid.
