# Supplementary material for: Detection of Pathological Markers of Neurodegenerative Diseases following Microfluidic Direct Conversion of Patient Fibroblasts into Neurons
Source: Int J Mol Sci. 2022 Feb 15;23(4):2147. doi: 10.3390/ijms23042147 (PMC8879457; doi:10.3390/ijms23042147)
Supplement: Supplementary file 1 [file ijms-23-02147-s001.zip › ijms-1553316-supplementary.pdf]

**Supplementary Information**

# Detection of pathological markers of neurodegenerative diseases following microfluidic direct conversion of patient fibroblasts into neurons

Cristiana Mollinari [1,2], Chiara De Dominicis [1,3], Leonardo Lupacchini [4], Luigi Sansone [4], Davide Caprini [5], Carlo Massimo Casciola [6], Ying Wang [7], Jian Zhao [7], Massimo Fini [4], Matteo Russo [4,8], Enrico Garaci [4,8], Daniela Merlo [1†]

Figure S1.
Figure S2.
Table S1.
Table S2.

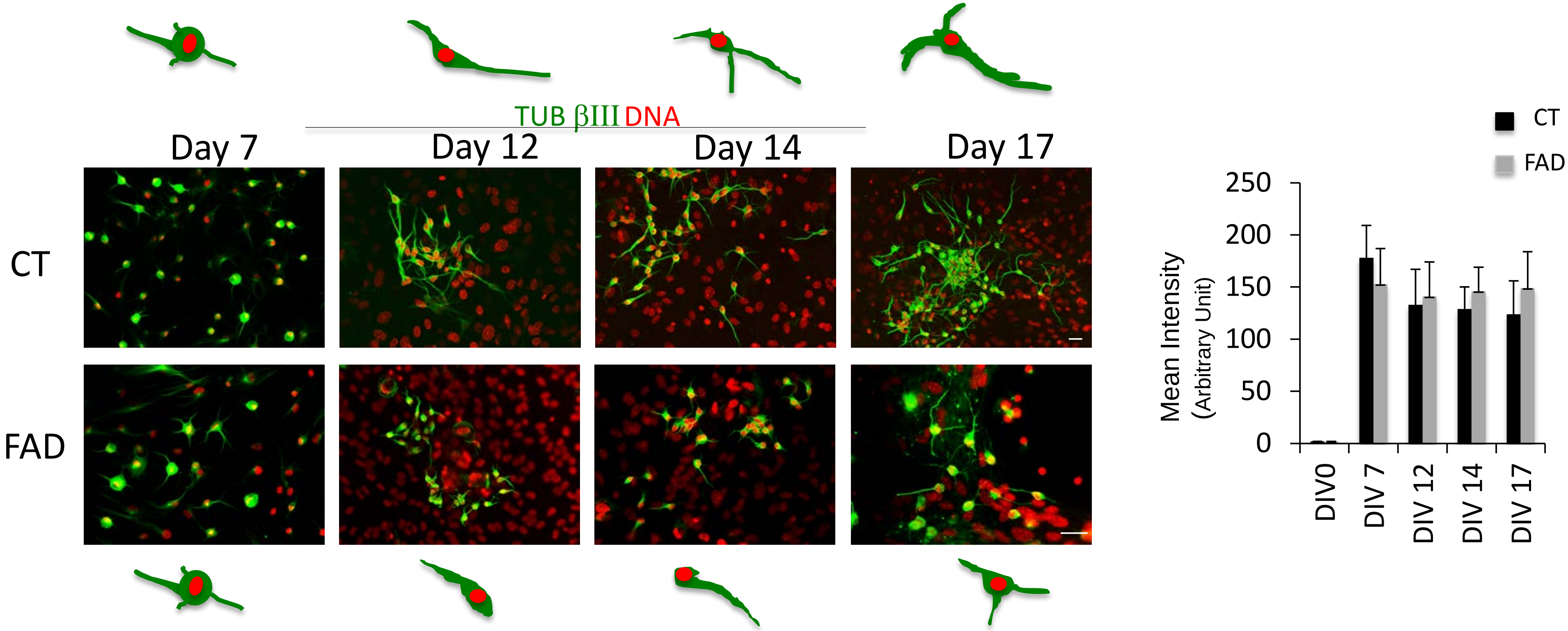


**Figure S1.** A panel of Immunofluorescence images showing a time-course of transdifferentiation of healthy fibroblasts and FAD fibroblasts induced to reprogram by chemical compounds (Hu et al., 2015). TUB βIII labelling intensity (green channel) appears strong in chemically induced cells soon after 7 days in culture, both in CT and FAD cells. Reprogrammed cells plated on glial monolayer maintain a high level of TUB βIII intensity (arbitrary unit) with no significative differences between CT and FAD, as shown in the histogram. Generally neurons tend to differentiate in groups and within the group, cells can show a different degree of maturation. In FAD cultures, it is possible to observe a significative reduction in neurite arborization and complexity along with a reduced number of cells with neuronal phenotype as compared with CT cultures. DNA is shown as red channel. Scale bar, 10 μm. Above and below the panel of immunofluorescence images, simplified drawings of differentiating ciNs during chemical reprogramming are shown to summarize that FAD cells display a lower neurite development and elongation suggesting a delay in neuronal maturation.

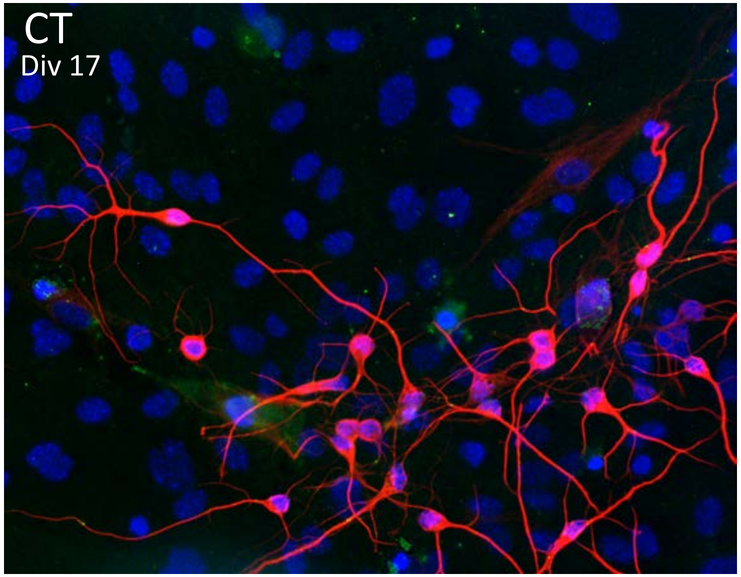


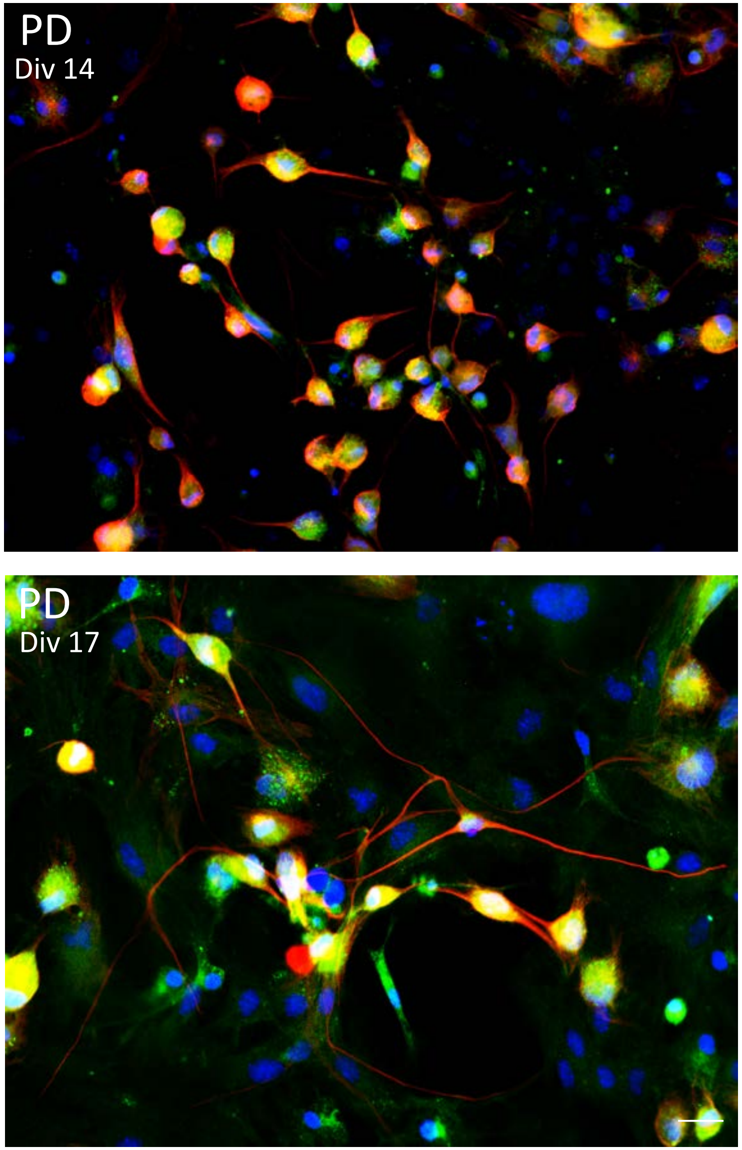


**Figure S2.** Immunofluorescence images of a cellular field from CT and PD cells at lower magnification after chemical transdifferentiation. A strong α-Synuclein labelling (green channel) is evident in the high majority of TUB βIII cells (red channel) from PD as compared with CT cells. DNA is shown as blue channel. Scale bar, 10 μm.

| Size of Mitochondria | Normal | | Elongated | | Elongated and Distorted | | Swollen with less dense matrix | | Swollen with severe loss of cristae with an empty matrix | |
|---|---|---|---|---|---|---|---|---|---|---|
| | Average | Stand dev | Average | Stand dev | Average | Stand dev | Average | Stand dev | Average | Stand dev |
| Lenght µm | 0,521 | 0,08 | 1,011 | 0,19 | 0,916 | 0,15 | X | X | X | X |
| Diameter µm | 0,212 | 0,04 | X | X | X | X | 0,527 | 0,09 | 0,776 | 0,12 |

| | Normal | Elongated | Elongated and Distorted | Swollen with less dense matrix | Swollen with severe loss of cristae with an empty matrix | Abnormal total |
|---|---|---|---|---|---|---|
| 7 div. CT | 90% | 6,71% | 1,90% | 0,93% | 0,46% | 10% |
| 7 div. FAD | 66% | 19,40% | 4,42% | 6,08% | 4,10% | 34% |
| 14 div. CT | 86% | 10,60% | 1,93% | 0,98% | 0,49% | 14% |
| 14 div. PD | 49% | 15,31% | 10,20% | 17,85% | 7,64% | 51% |

**Table S1** The percentage values of the different mitochondrial abnormalities observed by TEM analysis are indicated for each cellular group.

| Small-molecule compounds used | | | |
|---|---|---|---|
| **Name** | **Function(s)** | **Final Concentration (µM)** | **Company** |
| A83-01 | TGFβ inhibitor | 1 µM | ToCris |
| CHIR99021 | GSK-3 inhibitor | 3 µM | Axon MedChem |
| DAPT | γ-secretase inhibitor | 2 µM | Selleck |
| Dorsomorphin | BMP inhibitor | 1 µM | Cayman |
| Forskolin | Adenylyl cyclase activator | 10 µM | Sigma Aldrich |
| GO6983 | PKC inhibitor | 5 µM | Selleck |
| ISX9 | Neurogenic Modulator | 10 µM | Selleck |
| LDN193189 | TGF-beta/Smad inhibitor | 0.5 µM | Axon MedChem |
| P7C3-A20 | Neurogenic Modulator | 3 µM | Selleck |
| PD0325901 | MEK inhibitor | 1 µM | Selleck |
| Purmorphamine | Hedgehog agonist | 1 µM | Selleck |
| Repsox | TGFβ inhibitor | 1 µM | BioVision |
| RG108 | DNA methyltransferase inhibitor | 10 µ M | Selleck |
| SP600125 | JNK1/2/3 inhibitor | 10 µ M | Sigma Aldrich |
| VPA | histone acetyltranferases inhibitor | 500 µM | Calbiochem |
| Y27632 | ROCK1 inhibitor | 5 µM | Sigma Aldrich |
| db-cAMP | 2'-O-Dibutyryl-cAMP | 100 µM | PanReac |

**Table S2.** A list of the small molecules used in the chemical coktails of fibroblasts transdifferentiation for large and small scale experiments. The final concentration and the targets of the compounds are indicated.
